# The influence of text messages and anxiety on pain perception and its impact on orthodontic patients routine

**DOI:** 10.1590/2177-6709.25.5.030-037.oar

**Published:** 2020

**Authors:** Daniela Lasmar de Mendonça, Renata Rodrigues Almeida-Pedrin, Nayara Caldas Pereira, Paula Vanessa Pedron Oltramari, Thaís Maria Freire Fernandes, Ana Cláudia de Castro Ferreira Conti

**Affiliations:** 1 Centro Universitário do Norte, Escola da Saúde (Manaus/AM, Brazil).; 2 Universidade do Norte do Paraná, Departamento de Ortodontia (Londrina/PR, Brazil).; 3 Instituto Agenor Paiva de Pós-Graduação - FUNORTE/IAPPEM (Salvador/BA, Brazil).

**Keywords:** Orthodontics, Pain, Anxiety, Visual analogue scale

## Abstract

**Objective:**

This prospective study aimed at assessing the effects of anxiety and a follow-up text message on pain perception after the installation of fixed orthodontic appliances and its impact on the patients’ routine.

**Methods:**

The sample of this study consisted of 103 orthodontic patients, 40 males and 63 females (mean age 20.5 years), distributed in two groups: G1 (n=51), including control patients that did not receive any post-procedure communication; and G2 (n=52), including patients that received a structured text message. In baseline phase, the patients completed a questionnaire to assess their level of anxiety prior to treatment. Pain was assessed by using 100-mm visual analog scale (VAS) in baseline and ten times prospectively in predetermined time points. VAS was also applied to assess the patient’s routine alterations caused by the pain. All data were analyzed using ANOVA, Tukey, Mann-Whitney, *t*-test, chi-square and Spearman’s correlation tests. All statistical tests were performed with significance level of 5%.

**Results:**

Low-level and high-level anxiety was observed in 42.7% and 7.8% of the patients, respectively. Statistically significant correlation was observed between anxiety and pain (*p*< 0.05). Maximum mean pain intensity was detected in the second treatment day (G1=36.9mm and G2=26.2mm) and was significantly higher in G1. Nearly 53% of the patients in G1 reported alterations in the routine (18.8mm), while in G2 the percentage rate reached 28.8% (9.9mm) (*p*=0.013).

**Conclusions:**

Anxious patients report more pain after the installation of orthodontic appliances. Text messages were effective to reduce pain levels and to decrease the negative effects on patients’ daily routine.

## RESUMO

**Objetivo:**

O presente estudo prospectivo teve como objetivo avaliar os efeitos da ansiedade e de mensagens de texto de acompanhamento na percepção da dor após a instalação de aparelhos ortodônticos fixos e seu impacto na rotina dos pacientes.

**Métodos:**

A amostra deste estudo foi composta por 103 pacientes ortodônticos, 40 homens e 63 mulheres (idade média de 20,5 anos), distribuídos em dois grupos: G1 (n = 51), que incluiu pacientes controle, que não receberam comunicação pós-procedimento; e G2 (n = 52), incluindo os pacientes que receberam mensagem de texto estruturada. Na fase inicial, os pacientes responderam a um questionário para avaliar seu nível de ansiedade antes do tratamento. A dor foi avaliada por meio de escala visual analógica (EVA) de 100 mm antes da instalação dos aparelhos e em 10 períodos consecutivos predeterminados. A EVA também foi aplicada para avaliar as alterações de rotina do paciente causadas pela dor. Os resultados foram analisados usando ANOVA, Tukey, Mann-Whitney, teste t, Qui-quadrado e testes de correlação de Spearman. Todos os testes estatísticos foram realizados com nível de significância de 5%.

**Resultados:**

Níveis baixos e altos de ansiedade foram observados em 42,7% e 7,8% dos pacientes, respectivamente. Foi observada correlação estatisticamente significativa entre ansiedade e dor (*p*< 0,05). A intensidade média máxima da dor foi detectada no segundo dia de tratamento (G1 = 36,9mm e G2 = 26,2mm) e foi significativamente maior no G1. Quase 53% dos pacientes do G1 relataram alterações na rotina (18,8mm), enquanto no G2 o percentual atingiu 28,8% (9,9mm) (*p*= 0,013).

**Conclusões:**

Pacientes ansiosos relatam mais dor após a instalação de aparelhos ortodônticos. As mensagens de texto foram eficazes para reduzir os níveis de dor e diminuir os efeitos negativos na rotina diária dos pacientes.

## INTRODUCTION

Pain is usually experienced in the beginning of the orthodontic treatment.^1^ Most of the pain reported is related to lesions in the oral mucosa due to local trauma. Moreover, orthodontic forces applied for tooth alignment also play an important role in promoting pain.^2^ Usually, 91% of the orthodontic patients report discomfort, sensibility and pain during the installation of orthodontic appliances, while 39% report the same complaints after the orthodontic activation.^3^ The highest levels of pain are manifested in the second and third treatment days. The pain may reduce progressively after a few days, but it can persist months after installation, especially when a new archwire is installed.^5^

Aside from the physical complaints, psychological aspects, such as cognition, socialization and personality^6,7^ may also be affected by the increase in pain.^3^ Among those, anxiety emerges as a key aspect.^6,8^ Combined with anxiety, the increased perception of pain may hamper social and daily activities, especially eating and sleeping. Consequently, patients may respond by self-medicating^3^ and perhaps asking to quit the orthodontic treatment.^8-11^ This harmful combination could be attributed to specific dental stimuli that may trigger severe levels of anxiety that interfere with the orthodontic treatment.^12^

The search for strategies to reduce or eliminate anxiety in patients may contribute to decreasing their perception of pain.^13^ ^,^ ^14^ Phone calls^13,15,16^ and text messaging^8^ ^,^ ^17^ during the treatment have demonstrated positive outcomes for controlling anxiety^6^ and reducing the perception of pain.^8,13,17^ It has been reported that after dentoalveolar surgery, most patients are satisfied with telephone follow-up, and it was suggested that this type of follow-up procedure should always be done, both in public and private health services^15^ ^,^ ^16^. Keith et al.^17^ sent structured text messages to their patients explaining the potential alterations in routine and discomforts inherent to the orthodontic treatment. The authors observed that higher anxiety levels were detected within patients that did not receive the messages. Together with high anxiety, the patients also expressed higher perception of pain. 

Despite the potential association between anxiety and the perception of pain, there is no study dedicated to validate the impact of those variables in the daily routine of patients - especially in the early stage of orthodontic treatment. In practice, the impact of pain perception on patient’s motivation and compliance during the treatment is still unclear. 

The present study aimed at assessing the association between pain and anxiety in young adult patients after the installation of fixed orthodontic appliances. The hypothesis that text messages could contribute to decrease the pain associated with orthodontic treatment and its impact on patients’ daily routine was also investigated.

## MATERIAL AND METHODS

This prospective study was performed after the approval of the Committee of Ethics in Research of *Centro Universitário Unisagrado* (CAAE 53269815.5.0000.5502). All the patients (or their legal guardians) signed an informed consent form to participate in the study.

The patients were recruited from private dental offices between January 2016 and April 2017. According to the inclusion criteria the patients should be aged 14-30 years old, have permanent dentition with malocclusion and moderate crowding - justifying orthodontic treatment with fixed appliances -, should have access to a smartphone, and good oral health condition. Additionally, the patients that were included in the study had complete orthodontic charts that consisted of clinical records, dental casts, panoramic and cephalometric radiographs, and intra- and extraoral photographs. The exclusion criteria consisted of previous orthodontic treatment, medical history of chronic diseases and chronic self-medication, especially anxiolytic drugs. 

Power analysis was performed for sample size calculation with a significance level of 5% and test power of 80%. The standard deviation of 17.64 was adopted following the outcomes of Keith et al.^17^ According to these findings, a sample of 50 subjects would be necessary in each study group to support a difference of 10mm in the Visual Analogue Scale (VAS). Based on the calculation, the sample size was set at 103 patients. 

The sample was randomly divided into two groups. A computer-generated randomization list was created using Excel (2007, Microsoft Windows). Group 1 (G1, control) consisted of 51 patients (19 males and 32 females) aged between 14.1 and 30 years (mean age: 21.2 years). Group 2 (G2) consisted of 52 patients (21 males and 31 females) aged between 14.2 and 29.11 years (mean age: 19.9 years). G1 started orthodontic treatment not receiving any post-procedure communication, while G2 received these messages via SMS or WhatsApp Messenger (WhatsApp Messenger Inc., Mountain View, California, USA). The communication through messages (only one time) was established right after the fixed orthodontic appliances were installed.

The fixed orthodontic appliances were installed in all patients from permanent maxillary right first molar to the left maxillary first molar. The initial orthodontic archwire was 0.012-in or 0.014-in nickel-titanium (NiTi). Archwires for leveling were attached with individual elastomeric rings. Before treatment, a single orthodontist applied the Modified Corah Dental Anxiety Scale (MDAS) to patients and they completed the VAS for pain perception, during the orthodontic appointment. MDAS assessed the level of anxiety reported by the patient during the treatment. Scores below 5 indicated very low level of anxiety; scores between 6 and 10 indicated low level; scores between 11 and 15 indicated moderate level; and scores between 16 and 20 indicated extreme anxiety.^18^

Pain was assessed by using 100-mm VAS at baseline and ten times in a follow-up period along predetermined time points: T_0_) before the installation of appliances; T_1_) immediately after the installation; T_2_) 8 hours after the installation; T_3_) 24 hours after the installation; and daily up to the 7^th^ day (from T_4_ to T_9_). The last assessment was scored in the 14^th^ day (T_10_). All patients received the VAS form to fill at home at the first orthodontic appointment.

For the G2 individuals, text messages were sent once to the patients right after the first appointment, in order to improve their motivation, clarify the treatment approach and ask about their well-being in the early stage of orthodontic treatment.^17^

In the first clinical appointment after the 14^th^ treatment day, the patients answered a questionnaire that registered their perception of pain and the eventual use of analgesics during the treatment. A new VAS was applied to assess the potential alterations in the daily routine of patients, related to the experienced pain. The text messages were sent by the same researcher, as well as the collection and assessment of all data.

## STATISTICAL ANALYSIS

The comparison of pain perception among different groups and timings was performed using two-way ANOVA test with repeated values for timing. For multiple comparisons, Tukey test was applied. For the comparison of groups considering MDAS, Mann-Whitney test was used. Independent *t*-test was applied to compare groups and indicate if the treatment affected or not the daily routine of patients. Chi-square test associated the use of analgesics and the alterations in the routine of patients. The outcomes of MDAS and VAS were compared with Spearman’s correlation coefficient. All the statistic tests were performed with Statistica (StatSoft Inc., Tulsa, USA) software package version 13, with significance level set at 5%.

## RESULTS

The subjects included in the present study (n=103) were divided into two groups paired by age and sex. Chi-square and *t*-tests showed no statistically significant differences between groups, confirming sample pairing (*p*=0.74 and *p*=0.14, respectively).

According to MDAS, low-level anxiety was observed in 42.7% of the patients (G1=21; G2=23). Extreme anxiety was observed only in 7.8% (G1=5; G2=3) of the patients. The same tendency regarding anxiety results was found when the groups were analyzed separately (Table 1). Mann-Whitney test (p=0.259) indicated no statistically significant differences in the anxiety rates reported in both groups.

**Table 1:** Distribution of the anxiety rates scored based on Corah’s scale (MDAS) in Groups 1 and 2.

MDAS	G1		G2	
	n	%	n	%
Very low level of anxiety	12	23.5	16	30.8
Low level of anxiety	21	41.2	23	44.2
Moderate anxiety	13	25.5	10	19.2
Extreme anxiety	5	9.8	3	5.8
Total	51	100.0	52	100.0

G1: group 1; G2: group 2; According to Mann-Whitney test, statistically significant differences were not observed between groups (p = 0.259).

**Figure 1:**
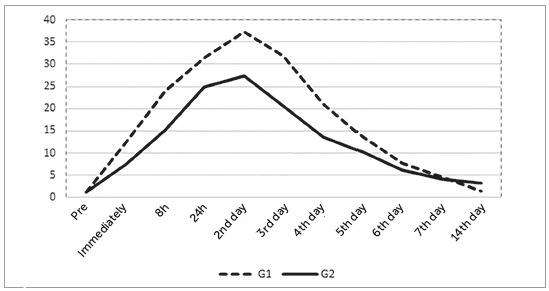
Mean values of pain perception measured in VAS throughout the evaluated time points.

To assess the level of pain, the patients were requested to complete the VAS before and after the installation of fixed appliances (T_0_ and T_1_, respectively). Patients with pain before the installation were replaced in the sample. In G1, the mean pain level observed immediately after the appliance placement was 10.8mm, while in G2 it was 7.2mm (Table 2). Comparisons of pain levels between groups in each time point were performed (from T_0_ to T_10_). This procedure enabled to evaluate if the text messages sent to Group 2 were efficient in reducing the perception of pain. The G2 reached lower pain level than G1 in all time points, except in the T_10_. Table 2 and Figure 1 show that patients in G1 and G2 reported higher scores for pain in the 2nd treatment day (G1=36.9 ± 3.1 mm; G2=26.2 ± 3.2 mm), and lower scores in the 14^th^ day (G1=1.2 ±1.2 mm; G2=2.9 ± 1.5 mm). Statistically significant differences between groups were observed within 8 hours after treatment, as well within the 2^nd^, 3^rd^ and 4^th^ treatment days. 

**Table 2:** Mean (mm) and standard deviation observed for the perception of pain assessed with VAS, distributed in relation to period.

Period	G1		G2	
	mean	SD	mean	SD
Pre (T_0_)	0.0	0.0	0.0	0.0
Immediately (T_1_)	10.8	1.7	7.2	1.8
8h (T_2_)	21.6*	2.5	14.3	1.8
24h (T_3_)	32.1	2.7	24.3	3.0
2nd day (T_4_)	36.9*	3.1	26.2	3.3
3rd day (T_5_)	29.2*	3.1	19.0	2.6
4th day (T_6_)	19.1*	2.3	12.6	1.9
5th day (T_7_)	12.7	1.5	9.3	1.7
6th day (T_8_)	7.4	1.1	5.8	1.2
7th day (T_9_)	4.3	0.7	4.0	1.0
14th day (T_10_)	1.2	0.3	2.9	1.5

VAS: Visual Analogue Scale; ANOVA (F = 5.05; p = 0.027*); G1: group 1; G2: group 2; SD: standard deviation; *: statistically significant difference between groups (p<0.05); T_0_) before the installation of appliances; T_1_) immediately after the installation; T_2_) 8 hours after the installation; T_3_) 24 hours after the installation; T_4_ to T_9_) daily up to the 7th day after treatment; T_10_): 14^th^ day.

In order to investigate if the level of anxiety (MDAS) influences the pain perception (VAS), a correlation was performed among the different time points between groups. In all time points, statistically significant higher scores for pain perception were observed in patients with higher levels of anxiety (*p*<0.05), except for T_3_ in G2 (*p*>0.05) (Table 3).

**Table 3:** Correlation between the outcomes of anxiety (MDAS) and pain perception (VAS).

Correlation	G1		G2		G1 + G2	
	r	p	r	p	r	p
MDAS x VAS T_1_	0.36	0.010*	0.35	0.010*	0.33	0.001*
MDAS x VAS T_2_	0.28	0.043*	0.27	0.054	0.31	0.001*
MDAS x VAS T_3_	0.35	0.011*	0.53	<0.001*	0.45	<0.001*
MDAS x VAS T_4_	0.39	0.005*	0.59	<0.001*	0.48	<0.001*
MDAS x VAS T_5_	0.35	0.013*	0.57	<0.001*	0.47	<0.001*
MDAS x VAS T_6_	0.29	0.037*	0.50	<0.001*	0.41	<0.001*
MDAS x VAS T_7_	0.29	0.043*	0.42	0.002*	0.38	<0.001*
MDAS x VAS T_8_	0.41	0.003*	0.47	<0.001*	0.45	<0.001*
MDAS x VAS T_9_	0.43	0.002*	0.36	0.008*	0.41	<0.001*
MDAS x VAS T_10_	0.35	0.013*	0.28	0.044*	0.29	0.004*

MDAS: modified scale for anxiety in Dentistry; VAS: visual analog scale; G1: group 1; G2: group 2; r: correlation coefficient; p: significance value; T_1_) immediately after the installation; T_2_) 8 hours after the installation; T_3_) 24 hours after the installation; T_4_ to T_9_) daily up to the 7^th^ day after treatment; T_10_): 14^th^ day; *: statistically significant correlation (p<0.05).

When the patients returned for appointment, they were asked about the use of analgesics for pain control. Nearly 36.9% (n=38) of the patients used analgesics during the study. Chi-square test showed a statistically significant difference (*p*=0.034) between groups. Most of the patients under analgesic drugs were found in Group 1 (n=24; 47.1%) (Table 4).

**Table 4:** Distribution of patients using analgesics in groups 1 and 2.

Group	Yes		No	
	n	%	n	%
G1	24	47.1	27	52.9
G2	14	26.9	38	73.1

Chi-square test (p=0.034).

Most patients (52.9%) stated that their routine was affected by the orthodontic treatment. Statistically significant outcomes were observed between groups. Patients in G1 were more affected by the orthodontic treatment in relation to G2 (*p*=0.013). When this alteration was quantified (VAS), G1 presented a mean value of 18.8 ± 2.2 mm and G2, of 9.9 ±1.5mm, a difference considered statistically significant (*p*=0.002), as seen in Table 5. 

**Table 5:** Distribution of G1 and G2 regarding the routine alterations by treatment, mean and standard error (SE) of the outcomes of VAS (visual analog scale) regarding the patients in groups 1 and 2 that reported their routine affected (T_11_).

G1		G2		p
n	%	n	%	
Routine alteration				
yes: 27	52.9	yes: 15	28.8	0.013*
no: 24	47.1	no: 37	72.1	
VAS (T_11_)				
mean	SE	mean	SE	0.002**
18.5	2.2	9.9	1.5	

* Chi-square. ** independent t test.

## DISCUSSION

The use of orthodontic appliances may trigger discomfort, especially in the early treatment phase when physical and psychological adjustments occur. Specific aspects, such as the severity of the malocclusion and patient’s age, may influence on the level of pain reported during treatment. In order to avoid age influence, only young patients (aged from 14 to 30 years) were included in this study. Regarding initial malocclusion, all patients presented discrete or moderate crowding. In these patients, the treatment started with 0.012-in or 0.014-in NiTi archwires (archwire set up was reported for methodological purposes, in particular, because the association of orthodontic archwire type and pain perception is not confirmed by the scientific literature).^19^ ^,^ ^20^

Considering the influence of sex on pain perception and also the fact that most of the patients were females (n=63), Groups 1 and 2 were compared regarding sex distribution and presented compatibility (*p*=0.74). This information is important to confirm the homogeneity of the groups and to reduce the risk of bias from variables that could influence the outcomes. This procedure is justified in other scientific studies that indicated more expressive pain perception in females compared to males.^7^ ^,^ ^21^ ^,^ ^22^

On the other hand, pain is not exclusively associated with physical stimuli, but also related to cognitive and emotional aspects. Anxiety is pointed to as the main psychological aspect related to the perception of pain.^8^ According to MDAS, most of the patients (42.7%) classified themselves within a low level of anxiety. Yet the level of anxiety was similarly distributed between groups (*p*=0.259) (Table 1). This outcome suggested that both groups had similar anxiety rates before treatment. This aspect is extremely important when comparing both groups regarding pain intensity and patients’ behavior after receiving text messages.

According to the present study, patients with higher anxiety level are more prone to complain of intense pain (Table 3). The same outcome was observed for the analysis within groups. Beck et al^20^ found similar results with the application of MDAS and the State-Trait Anxiety Inventory (STAI). The authors observed statistically significant findings, showing that higher anxiety rates lead to higher pain perception levels. Similarly, Bergius et al^21^ observed that despite the lack of statistically significant differences between the anxiety levels, patients more anxious reported higher pain scores in the VAS.

Considering anxiety as a contributing factor to pain experience,^23^ patient-management strategies, such as text messaging, play an important part controlling anxiety and reducing the perception of pain. Compared to G1, G2 presented lower values for the perception of pain along all time points (Table 2 and Fig 1). However, the patients in both groups referred more pain in the 2^nd^ treatment day (Group 1 = 36.9 ± 3.1 mm; Group 2 = 26.2±3.2mm), while less pain was referred in the 14^th^ treatment day (Group 1 = 1.2± 1.2mm; Group 2 = 2.9±1.5mm). Statistically significant differences were observed between groups within 8 hours after the installation of the orthodontic appliances, as well within 2, 3 and 4 days. Accordingly, Bartlett et al^13^ showed that a single phone call after the installation of fixed appliances reduced the level of anxiety and consequently the related perception of pain. Similar findings were observed by Keith et al^17^ and Cozzani et al.^8^ These authors sent text messages to the patients for 7 days^17^ and assessed the efficiency of text messaging and phone calls for controlling pain^8^, respectively. These studies highlight the importance of communication in Orthodontics (via phone calls, text messaging, and e-mails), which includes providing information about treatment progress, instructions about potential discomfort, and encouraging messages.^8^ ^,^ ^13^ ^,^ ^17^

According to Table 4, in G1 nearly half (47.1%) of the patients considered analgesics necessary for pain control, while in G2 less patients had the same opinion (26.9%). This outcome confirms that text-messaging could reduce the perception of pain and the consequent need for analgesics. The use of analgesics is common in the early phase of orthodontic treatment.^24^ Hence, any approach, such as text-messaging, to decrease the use of medication may benefit patients. These findings corroborate the studies of Cozzani et al^8^ and Johal et al,^25^ which observed that most of the patients under analgesic medication did not receive text messages. In their studies, statistically significant difference between groups was observed in the first day of investigation. Bergius et al^21^ reported, more specifically, that the use of analgesics is more common in the first day after the installation of fixed appliances in women.

Possibly, the present study is the only one to investigate the potential impact of pain in the routine of orthodontic patients. Forty-two (40.77%) patients complained about routine alterations due to the pain experienced during the initial phase of treatment. The comparison between groups revealed statistically significant differences. In particular, patients in G1 had their routine more affected (52.9%) by the pain than the patients in G2 (28.8%), as seen in Table 5. When patients that reported routine alterations quantified how much impact in their lives was caused by the pain experience (VAS), statistically significant differences (*p*=0.002) were also observed between groups. G1 presented a mean value of 18.8±2.2mm and G2, of 9.9±1.5mm (Table 5). In this context, patients that did not receive text messages presented more discomfort and alteration in the daily routine. Clarifying the potential positive and negative experiences during the orthodontic treatment, as well as asking about the patient well-being through text messaging, may influence the treatment success. In practice, it is important because optimal treatment outcomes depend on strategies to minimize discomfort and pain experienced by the patients.^17^ Comfort and quality of life during the daily routine may motivate the patient towards a more collaborative attitude during the orthodontic treatment.^26^

The present study provided relevant data regarding pain perception and the level of anxiety in orthodontic patients after the installation of fixed appliances. As a limitation of the study, the patients were evaluated only 14 days after the installation of orthodontic appliances. In future research, we may consider sending text messages or phone calls follow-up for a longer period of evaluation. Based on the findings, the present study suggests the use of text messages to explain and clarify the treatment progress in order to minimize the perception of pain in the early phase of orthodontic treatment.

## CONCLUSION

» Anxious patients presented higher pain levels during initial phase of orthodontic treatment.
» When the perception of pain was compared between groups, significantly reduced pain was perceived by patients within 8 hours after appliance installation, as well within the 2^nd^, 3^rd^ and 4^th^ treatment days when they received the post-procedure message.
» A significant difference in routine alteration was observed between groups, patients that were not contacted with text message had their routine affected twice as much due to the pain experienced.
